# 3D-EAUS and MRI in the Activity of Anal Fistulas in Crohn's Disease

**DOI:** 10.1155/2016/1895694

**Published:** 2015-12-27

**Authors:** Maria Eleonora Alabiso, Francesca Iasiello, Gianluca Pellino, Aniello Iacomino, Luca Roberto, Antonio Pinto, Gabriele Riegler, Francesco Selvaggi, Alfonso Reginelli

**Affiliations:** ^1^Department of Radiology, Ascalesi Hospital, 80100 Naples, Italy; ^2^Department of Internal and Experimental Medicine, Second University of Naples, 80100 Naples, Italy; ^3^Unit of Colorectal Surgery, Department of Medical, Surgical, Neurologic, Metabolic and Ageing Sciences, Second University of Naples, 80100 Naples, Italy; ^4^Department of Radiology, Cardarelli Hospital, 80100 Naples, Italy; ^5^Unit of Gastroenterology, Department of Medical, Surgical, Neurologic, Metabolic and Ageing Sciences, Second University of Naples, 80100 Naples, Italy

## Abstract

*Aim.* This study aspires to assess the role of 3D-Endoanal Ultrasound (3D-EAUS) and Magnetic Resonance Imaging (MRI) in preoperative evaluation of the primary tract and internal opening of perianal fistulas, of secondary extensions and abscess. *Methods.* During 2014, 51 Crohn's disease patients suspected for perianal fistula were enrolled. All patients underwent physical examination with both the methods and subsequent surgery. *Results.* In the evaluation of CD perianal fistulas, there are no significant differences between 3D-EAUS and MRI in the identification of abscess and secondary extension. Considering the location, 3D-EAUS was more accurate than MRI in the detection of intersphincteric fistulas (*p* value = 10^−6^); conversely, MRI was more accurate than 3D-EAUS in the detection of suprasphincteric fistulas (*p* value = 0.0327) and extrasphincteric fistulas (*p*  value = 4 ⊕ 10^−6^); there was no significant difference between MRI and 3D-EAUS in the detection of transsphincteric fistulas. *Conclusions.* Both 3D-EAUS and MRI have a crucial role in the evaluation and detection of CD perianal fistulas. 3D-EAUS was preferable to MRI in the detection of intersphincteric fistulas; conversely, in the evaluation of suprasphincteric and extrasphincteric fistulas the MRI was preferable to 3D-EAUS.

## 1. Introduction

Perianal fistula is a chronic inflammatory condition defined as an abnormal perianal tract that connects two epithelial surfaces, usually the anal canal and the perianal skin [1]. This condition is often highly recurrent and may require repeated surgical treatments [2, 3]. Perianal fistulas predominantly affect young males with a male-to-female ratio of 2 : 1 [2, 4]. The most common symptom is discharge, but local pain is also frequent [3]. Patients suffering from Crohn's disease (CD) often experience perianal disease and have complex perianal sepsis requiring repeated treatments [5]. Surgery still plays a relevant role in perianal CD, with a significant prevalence of recurrence [4]. Accurate preoperative assessment of a fistula and its complications, such as secondary extensions or abscess, is mandatory to perform a successful surgery [6] while preserving anal function and continence, but no single tool can effectively depict the anatomy of perianal fistulas in these patients. Overly aggressive fistulotomy can lead to postoperative fecal incontinence, whereas inappropriate conservative treatment could lead to fistula recurrence [5]. Preoperative imaging modalities can alert the surgeon to fistula characteristics that might otherwise be missed. Aim of this study is to assess the role of 3D-Endoanal Ultrasound (3D-EAUS) and Magnetic Resonance Imaging (MRI) in diagnosing primary tract, and secondary extension, in localizing internal opening of perianal fistulas and identifying the relation between the anal fistula and anal sphincters.

## 2. Methods

### 2.1. Study Design and Population

Between January 2014 and December 2014, 51 patients with known CD (37 M; 14 F; age range: 28–56 years; mean: 42 years), suspected for perianal fistula, were enrolled. All patients were referred to our department after a previous clinical diagnosis of perianal fistula and were preoperatively evaluated with 3D-EAUS and MRI and subsequently underwent surgery. In accordance with our institute guidelines, every patient received and signed written consent forms. The eventual diagnosis of the fistula anatomy was made after combining the findings of all of the modalities (3D-EAUS, MRI, and surgery).

#### 2.1.1. 3D-EAUS

The examinations were performed with a Bruel and Kjaer ProFocus system Ultra View-2202 (Mileparken 34, 2730 Herlev, Denmark) with a model 2050 transducer equipped with a double multifrequency crystal (range: 6–16 MHz), with 360° mechanical rotation at a speed of 1.9–2.8 rotations/s, focus range up to 45 mm, dimensions 550 × 270 × 40 × 17 mm, and automatic extraction and field depth up to 10 cm. All patients were examined in the lateral decubitus position without any prior bowel preparation and without any anesthesia. The transducer was covered with a condom and, after adequate lubrication, placed inside the anal canal. The transducer was firstly advanced as far as the rectal ampulla before continuing with more caudal scans; it was then automatically withdrawn to the superficial perianal plane. Images were viewed in planes perpendicular to the transducer, which was kept with the same orientation so that the anterior wall was always visualized at the 12 o'clock position, the left wall at 3 o'clock, the posterior wall at 6 o'clock, and the right wall at 9 o'clock.

Three scan planes were acquired:

1. The deeper plane corresponded to the proximal extremity of the anal canal, where there is the typical U-shaped sling appearance of the hyperechoic puborectalis muscle with the wider end towards the pubis.
2. The intermediate plane included the hypoechoic internal anal sphincter (IAS), the perianal body, and the transverse perianal muscle.
3. The superficial plane corresponded to the level of the distal extremity of anal canal and included the hyperechoic layer of the submucosal portion of the external anal sphincter (EAS).

All images were retrospectively analyzed by two observers (reader 1 and reader 2), who were unaware of the MRI results. The two observers evaluated the data independently of each other without knowing the test results. To avoid discrepancy, both observers examined the case together until agreement was reached.

Radiological examination aimed to determine the following fistula characteristics: (1) the primary tract, defined according to the criteria of Parks et al. [7] as intersphincteric, transsphincteric, extrasphincteric, or suprasphincteric; in the intersphincteric fistulas the submucosal fistulas were included, lying in the superficial submucosal plane lateral to the subcutaneous portion of the external anal sphincter; (2) the internal opening, localized with respect to a clock face as described above; and (3) secondary extension, including horseshoe tract and abscess formation. The anatomic location of any secondary extension arising from the primary fistula track was recorded as intersphincteric, ischiorectal, or supralevator. A horseshoe extension was defined as any extension from the primary track that appeared to extend to both sides of the internal opening, and such an extension was classified as intersphincteric or ischiorectal.

Fistula tracks were visualized as tube-like, hypoechoic lesions. The internal fistula opening was identified as a hypoechoic area in the intersphincteric plane, as a defect in the internal anal sphincter, or as a subepithelial breach that connected to the fistulous tract through an internal sphincter defect [8].

After the EAUS procedures, the characteristics of the fistula were classified according to the same criteria used in the clinical evaluation.

#### 2.1.2. MRI

MR imaging studies were performed on a 1.5 T closed magnet (Magnetom Symphony, Siemens, Germany). The patients were placed in the supine position.

T2-weighted turbo spin-echo (TSE) sequences (TR 5370, TE 126, averages 2, flip angle 150, slice thickness 4, bandwidth 130, and FOV READ 230 mm) were acquired in sagittal plane, which was used as reference to obtain para-axial planes (perpendicular to the anal canal) and paracoronal planes (parallel to the anal canal). In the para-axial planes the following sequences were acquired: T1-weighted TSE (TR 611 ms, TE 11 ms, averages 2, flip angle 150 deg, slice thickness 5 mm, bandwidth 130, and FOV READ 270 mm), T2-weighted TSE (TR 7710 ms, TE 114 ms, averages 2, flip angle 180 deg, slice thickness 3.5 mm, bandwidth 130, and FOV READ 334 mm), and T2-weighted Haste with and without suppression of fat signal (TR 9860 ms, TE 114 ms, averages 2, flip angle 180 deg, slice thickness 3.5 mm, bandwidth 130, and FOV READ 250 mm). In the paracoronal plane T2-weighted TSE with suppression of fat signal was acquired (TR 2500 ms, TE 104 ms, averages 2, flip angle 150 deg, slice thickness 4 mm, bandwidth 130, and FOV READ 300 mm).

Two radiologists (reader 3; reader 4) evaluated the images without knowing the results of the 3D-EAUS. Each component of the anal fistula was categorized and recorded using the similar criteria of 3D-EAUS. Fistula tracks were visualized as tube-like, hyperintense or hypointense lesions. The internal fistula opening was identified as a hyperintense or hypointense area in the intersphincteric plane, as a defect in the internal anal sphincter, or as a subepithelial breach that connected to the fistulous tract through an internal sphincter defect.

### 2.2. Statistical Methods

The statistical analyses were performed using Matlab statistical toolbox version 2008 (MathWorks, Natick, MA, USA) for Windows at 32 bits. McNemar's exact test [9] and *χ* ^2^ test with Yates correction [10] were performed to determine the higher accuracy between 3D-EAUS and MRI in the individualization of primary tract, according to Parks classification, and in the identification of secondary extensions and abscess.

In addition the sensitivity and specificity with confidence intervals at 95% [11] were defined for the diagnostic procedures. All tests with *p* value < 0.05 were considered as significant.

## 3. Results

All patients well tolerated the exam and there were no side effects reported. The analysis of radiological examinations of 51 patients, respectively, for MRI and 3D-EAUS, showed the presence of intersphincteric fistulas in 5.88% (3/51) of cases versus 45.10% (23/51), transsphincteric fistulas in 19.61% (10/51) versus 23.53% (12/51), suprasphincteric fistulas in 21.57% (11/51) versus 7.84% (4/51), and extrasphincteric fistulas in 41.18% (21/51) versus 5.88% (3/51) and absence of pathology in 11.76% (6/51) versus 17.65% (9/51) (Table 1).

**Figure 1:**
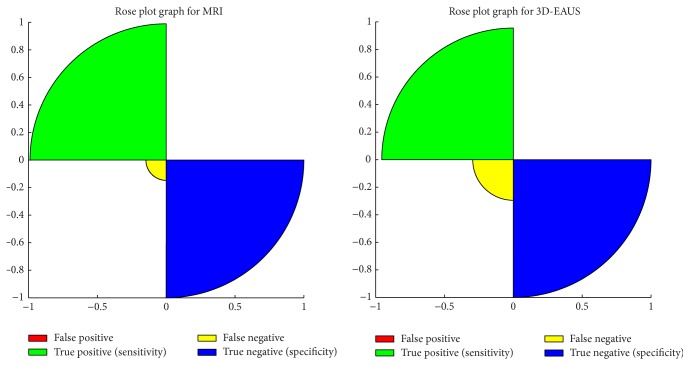
Rose plot graphs of sensitivity and specificity for MRI and 3D-EAUS.

There was no significant difference between MRI and 3D-EUAS (Table 2 and Figure 1) in specificity (100% versus 100%) and sensitivity (91.30%, with IC = 79.2%–97.6% versus 97.80%, with IC = 87.9%–100%) (Table 2). McNemar's exact test confirmed that there was no significant difference between MRI and 3D-EAUS in the evaluation of patients with pathology (*p* value = 0.187). Considering each location, according to Parks classification, 3D-EAUS result was more accurate than MRI in the detection of intersphincteric fistulas (*p*  value = 10^−6^); conversely, MRI was more accurate than 3D-EAUS in the detection of suprasphincteric fistulas (*p* value = 0.0327) and extrasphincteric fistulas (*p*  value = 4 ⊕ 10^−6^), while there was no significant difference between MRI and 3D-EAUS in the detection of transsphincteric fistulas.

In Table 3 we showed the different accuracy between MRI and 3D-EAUS in the identification of primary tract, secondary extension, and abscess, considering that one patient could be affected from more symptoms too. The *χ* ^2^ test with Yates correction showed that in the evaluation of primary tract, secondary extension, and abscess there were no significant differences between MRI and 3D-EAUS (58.82% versus 52.94% with *p* value = 0.55; 86.27% versus 80.39% with *p* value = 0.42; 15.69% versus 5.88% with *p* value = 0.11, resp.). Concerning secondary extensions, there were 27 patients (61.4%) with concomitant abscesses and 17 (38.6%) with horseshoe extension. No differences were observed concerning detection of each of these findings between the two modalities (abscess, 27 versus 25, *p* = 0.15; horseshoe track, 17 versus 16, *p* = 0.31 MRI versus 3D-EAUS).

## 4. Discussion

Anal fistulas are a significant cause of morbidity associated with a severe reduction of quality of life. It represents a common clinical problem affecting approximately 0.01% of the general population, predominantly young adults, and, differently from pelvic floor disorders, afflicts men two times more often than women [2, 4, 12]. Up to 60% of CD patients have perianal disease, of whom 30% have perianal fistula [5]. Ten percent of CD can have perianal fistula as first presenting symptom, before receiving CD diagnosis.

Anal fistula is defined by an abnormal perianal tract that connects two epithelized surfaces: the anal canal to the perianal skin. Some fistulas have a tendency to recur, despite seemingly curative surgery. Recurrence is usually due to infection that has gone undetected and untreated [1]. The most common symptom is discharge (65% of the cases), but local pain due to inflammation is also common. Perianal fistulas may be caused by several inflammatory conditions and events, including CD [13, 14]. The aetiology of perianal disease in CD is debated, and no single factor can be identified as responsible of subsequent anorectal sepsis, probably resulting from a combination of microbiological, immunological, and genetic factors [5]. The most widely used one is Parks et al. classification system that was derived from analysis of 400 consecutive patients referred for specialist evaluation of perianal fistulas. Parks et al. [7] classified fistulas into four main groups. Intersphincteric fistulas were the most commonly noted (45%) and are characterized by a primary tract that courses in the intersphincteric space without penetrating the external sphincter. In the intersphincteric fistulas we included the submucosal fistulas, lying in the superficial submucosal plane lateral to the subcutaneous portion of the external anal sphincter; transsphincteric fistulas were slightly less common (30%) and traverse the external sphincter and pass into the ischioanal fossa, below the level of the puborectalis muscle. Suprasphincteric fistulas (20%) extend within the intersphincteric plane superior to the puborectalis before penetrating the levator musculature to course within the ischioanal fossa. Extrasphincteric fistulas (5%) course within the ischioanal fossa and penetrate the levator musculature without traversing either the internal or the external sphincters opening directly into the rectum [4, 7]. All of these fistula types may be complicated by abscesses and by secondary tracks. In addition fistulas can spread circumferentially in the intersphincteric space, ischioanal fossa, or supralevator space. Circumferential branches or abscesses that extend on both sides of the interior opening are known as horseshoe branches or abscesses [7].

Incorrect classification and/or determination of extent increases risk of incomplete healing, recurrent fistula, and inadvertent sphincter injury.

3D-EAUS is a valuable tool to represent the normal anatomy of the anal canal and it is simple, cheap, readily available, less demanding for the patient, and with high diagnostic accuracy. It allows rapid evaluation for specialized equipment, is easy to perform and easily reproducible and painless, and does not require patient preparation.

**Figure 2:**
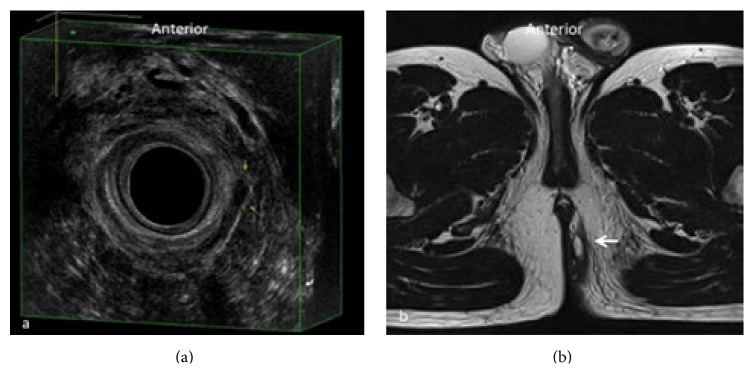
Submucosal fistula in the superficial plane corresponding to the level of the distal extremity of anal canal. In (a) 3D-EAUS, including the hyperechoic layer of the submucosal portion of the external anal sphincter (EAS), shows a submucosal fistula extending from 3 o'clock to 5 o'clock, lying external to the submucosal portion of the EAS (yellow arrows). The same plane on MRI (b), which could be avoided in this kind of fistulas (white arrow); 3D-EAUS is often sufficient as a preoperative diagnostic method.

**Figure 3:**
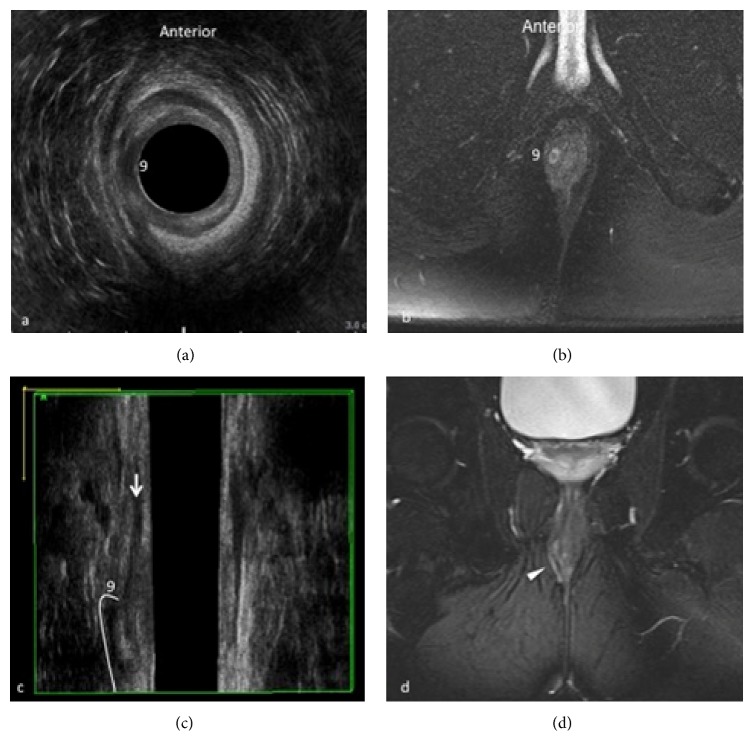
Intersphincteric fistula at 9 o'clock. 3D-EAUS demonstrates the proximal origin of the fistulous tract from the internal anal sphincter and its location in the intersphincteric plane on both axial (a) and coronal plane (c), better depicting the fistulous tract in the intersphincteric space than MRI (b, d).

It provides excellent imaging of the rectal wall, of the internal and external sphincters and of the intersphincteric plane, of muscle mobility, and of the position of the internal opening, essential for planning surgical approach to reduce the risk of incontinence. This method can be very useful also in the follow-up of anal diseases, both to study surgical drainages and in the postoperative study of anal fistulae. 3D-EAUS represents the first investigation in patients with perianal fistulas that allows real-time visualization; it has the potential to become the initial and most cost-effective investigation for fistula disease, which may alleviate the need for MRI in most patients. It is fully sufficient as a preoperative diagnostic method in most patients with intersphincteric and transsphincteric/submucosal fistula above all with single tract and without abscess, better depicting the intersphincteric plane and both the internal and external sphincters (Figures 2 and 3). 3D-EAUS has some limitations since it is highly operator dependent, it has limited ability to resolve ischioanal and supralevator infections, and it does not allow a reliable distinction between infection and fibrosis [4, 6, 15–18].

**Figure 4:**
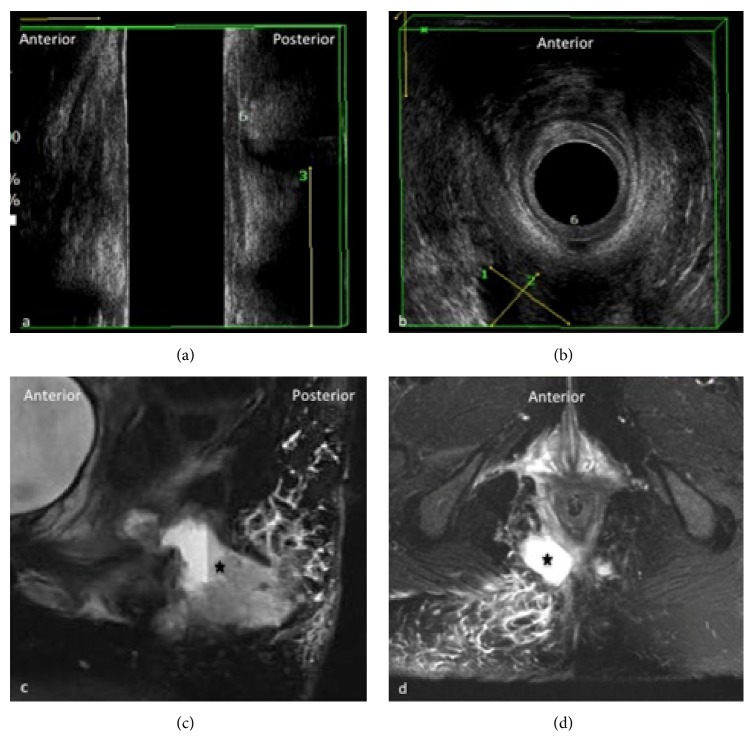
Extrasphincteric fistula at 6 o'clock with abscess. Sagittal (a) and axial (b) view of anal canal on 3D-EAUS showing at 6 o'clock (6) the proximal origin of an extrasphincteric fistula which drains into a big extrasphincteric abscess (calibers 1–3). On (c) and (d) sagittal and axial view, respectively, of anal and perianal region on MRI which is indispensable to demonstrate the complete extension of the extrasphincteric abscess (black star) and the appearance of edematous surrounding tissues.

MRI has the advantage of an excellent intrinsic soft-tissue resolution, thus showing the fistula tract in the context of the surrounding structures. It has a wider FOV than 3D-EAUS and it is more suited for the assessment of complex branching tracts, the lateral extension into the perianal spaces, and the cranial extension above the levator ani (Figure 4) [19, 20].

It is useful to improve treatment by correct assessment of the extent of disease, in the treatment response/monitoring of perianal fistulas, especially in CD; it is also valuable to differential diagnosis between infections from fibrosis, ischioanal and supralevator infections, and supra- and infralevator extension. It could be a valid second-level examination in case of abscesses or complex tracts and also through the pelvic diaphragm and finally where internal opening cannot be simply shown [20–25].

**Figure 5:**
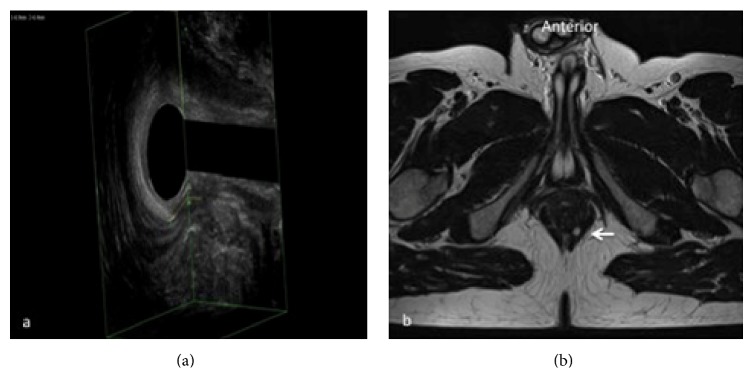
Transsphincteric fistula at 5 o'clock. Both 3D-EAUS (calibers) (a) and MRI (white arrow) (b) are accurate in the detection of transsphincteric fistulas. Thanks to 3D technique 3D-EAUS may show the entire extension of the fistula while on MRI it appears on two different planes.

In our series we were able to demonstrate that both MRI and 3D-EAUS can be used to assess transsphincteric fistulas. However, basing on 3D-EUAS exams alone, up to 14% of suprasphincteric fistulas can be overlooked or not correctly diagnosed (Table 1, Figure 5).

In conclusion, both EAUS and MRI have a crucial role in the evaluation and detection of perianal fistulas. 3D-EUAS is more accurate in comparison to MRI in the individuation of intersphincteric/submucosal fistulas, where it could be fully sufficient as a preoperative diagnostic method, better depicting the intersphincteric plane and both the internal and external sphincters. In fact, the introduction of 3D technique has optimized US evaluation. MRI is more accurate in comparison to 3D-EUAS in the individuation of suprasphincteric and extrasphincteric fistulas with the reported advantage of an excellent intrinsic soft-tissue resolution and higher panoramicity, thus showing the fistula track in the context of the surrounding structures. 3D-EUAS and MRI are statistically equivalent in the detection of transsphincteric fistulas and in the evaluation of abscess and secondary extension.

## Figures and Tables

**Table 1:** Type of perianal fistulas, according to Parks classification, observed with 3D-EAUS and MRI.

Location	3D-EAUS Number of patients (%)	MRI Number of patients (%)	Hypothesis	McNemar's exact test (*p* value)
Intersphincteric	23 (45.10)	3 (5.88)	MRI < 3D-EAUS	10^−6^
Transsphincteric	12 (23.53)	10 (19.61)	MRI < 3D-EAUS	0.344
Suprasphincteric	4 (7.84)	11 (21.57)	MRI > 3D-EAUS	0.0327
Extrasphincteric	3 (5.88)	21 (41.18)	MRI > 3D-EAUS	4 ⊕ 10^−6^
Absence of pathology	9 (17.65)	6 (11.76)	MRI < 3D-EAUS	0.187
Total	**51 (100)**	**51 (100)**	MRI > 3D-EAUS	

**Table 2:** Differences between MRI and 3D-EAUS.

Parameters	3D-EAUS		MRI	
	Value %	IC 95%	Value	IC 95%
Sensitivity	97.80	(87.9, 100.0)	91.11	(79.2, 97.6)
False negative	2.20	(2.0, 17.1)	8.89	(3.0, 20.2)
Specificity	100.00	(91.3, 100.0)	100.0	(91.3, 100.0)
False positive	0.00	(0.2, 6.8)	0.00	(0.2, 6.8)
Accuracy	98.00	(88.2, 100.0)	92.20	(80.3, 98.2)

**Table 3:** Proportion of positive patients to 3D-EAUS and MRI in the diagnosis of primary tract of anal fistulas, secondary extensions, and abscess and *χ* ^2^ test with Yates correction.

	MRI %	3D-EAUS %	Hypothesis	*χ* ^2^ test (*p* value)
Primary tract	58.82 (30)	52.94 (27)	MRI > 3D-EAUS	0.55
Secondary extension	86.27 (44)	80.39 (41)	MRI > 3D-EAUS	0.42
Abscess	15.69 (8)	5.88 (3)	MRI > 3D-EAUS	0.11
